# Incidence and Predictors of Maternal Cardiovascular Mortality and Severe Morbidity in the Netherlands: A Prospective Cohort Study

**DOI:** 10.1371/journal.pone.0056494

**Published:** 2013-02-14

**Authors:** Claartje M. Huisman, Joost J. Zwart, Jolien W. Roos-Hesselink, Johannes J. Duvekot, Jos van Roosmalen

**Affiliations:** 1 Department of Obstetrics and Gynaecology, Bronovo Hospital, The Hague, The Netherlands; 2 Department of Obstetrics, Leiden University Medical Centre, Leiden, The Netherlands; 3 Department of Obstetrics and Gynaecology, Deventer Hospital, Deventer, The Netherlands; 4 Department of Cardiology, Erasmus Medical Centre, Rotterdam, The Netherlands; 5 Department of Obstetrics and Gynaecology, Erasmus Medical Centre, Rotterdam, The Netherlands; 6 Department of Medical Humanities, EMGO-Institute for Health and Care Research, VU University Medical Centre, Amsterdam, The Netherlands; Université de Montréal, Canada

## Abstract

**Objective:**

To assess incidence and possible risk factors of severe maternal morbidity and mortality from cardiovascular disease in the Netherlands.

**Design:**

A prospective population based cohort study.

**Setting:**

All 98 maternity units in the Netherlands.

**Population:**

All women delivering in the Netherlands between August 2004 and August 2006 (n = 371,021)

**Methods:**

Cases of severe maternal morbidity and mortality from cardiovascular disease were prospectively collected during a two-year period in the Netherlands. Women with cardiovascular complications during pregnancy or postpartum who were admitted to the ward, intensive care or coronary care unit were included. Cardiovascular morbidity was defined as cardiomyopathy, valvular disease, ischaemic heart disease, arrhythmias or aortic dissection. All women delivering in the same period served as a reference cohort.

**Main outcome measures:**

Incidence, case fatality rates and possible risk factors.

**Results:**

Incidence of severe maternal morbidity due to cardiovascular disease was 2.3 per 10,000 deliveries (84/358,874). Maternal mortality rate from cardiovascular disease was 3.0 per 100,000 deliveries (11/358,874). Case fatality rate in women with severe maternal morbidity due to cardiovascular disease was 13% (11/84). Case fatality rate was highest in aortic dissection (83%). Pre-existing acquired or congenital heart disease was identified in 34% of women. Thirty-one percent of women were of advanced maternal age (>35 years of age) and 5 percent above 40 years of age. Possible risk factors for cardiovascular morbidity were caesarean section (either resulting in or as a result of cardiovascular disease), multiple pregnancy, prior caesarean section, non-Western ethnicity and obesity.

**Conclusions:**

In the Netherlands cardiovascular disease is a rare cause of severe maternal morbidity with an incidence of 2.3 per 10,000 deliveries and a high case fatality rate of 13%. Cardiovascular complications develop mostly in women not known with cardiac disease pre-pregnancy.

## Introduction

Cardiovascular disease complicates approximately 1–3% of pregnancies and is responsible for 10 to 15% of maternal mortality.[1]–[3] Since more women with congenital and acquired heart disease reach fertile age due to improved medical care, the incidence of cardiovascular disease in pregnancy is increasing.

The UK Confidential Enquiries into Maternal and Child Health have identified cardiovascular disease as an increasingly important cause of indirect maternal death. Increasing prevalence of advanced maternal age, obesity and pre-existing hypertension constitute risk factors for the development of cardiovascular disease during pregnancy. In the last two triennia ending in 2005 and 2008, cardiac disease was *the* most frequent cause of indirect maternal death with a maternal mortality rate (MMR) of 2.27 and 2,31 per 100,000 maternities, respectively.[3]

Although there was a decline in maternal deaths related to congenital heart disease (CHD) from 4.6 per million to 1.3 per million maternities in the triennia ending in 1996 and 2008, maternal mortality from acquired heart diseases (AHD) increased from 3.8 per million in 1996 to 21.8 per million maternities in 2008.[3]–[6]

In the Netherlands, cardiovascular disease was the main cause of indirect maternal mortality (MMR 1.6 per 100,000 live births) between 1993 and 2005, and significantly increased as compared to 1983 to 1992, when it was the second most frequent cause (MMR 0.6 per 100,000 live births; OR 2.5; 95% CI 1.4–4.6) after cerebrovascular disorders. Indirect maternal deaths are those resulting from previously existing disease or disease that develops during pregnancy, not due to direct obstetric causes, but which is aggravated by physiologic effects of pregnancy. When looking at all causes of maternal mortality, cardiovascular disease rose from the fourth to the second most frequent cause after (pre-) eclampsia.[7]

Pregnancy represents a major stress to the cardiovascular system, not only due to a 50% increase in blood volume and cardiac output and a decrease in systemic vascular resistance, but also due to structural changes in the vascular wall such as hypertrophy of vascular endothelium and smooth muscle and a higher risk of thrombosis. This explains why women with heart disease may experience deterioration of clinical function or even develop heart failure during pregnancy and have a higher complication rate.

In this nationwide study we aimed to investigate the incidence and possible risk factors of severe maternal morbidity from cardiovascular disease and its case fatality rate (CFR) during pregnancy, both in women with or without pre-existing heart disease.

## Methods

### Study Design

All cases of severe maternal morbidity and mortality were collected in a nationwide cohort study called the LEMMoN-study, conducted between August 1^st^ 2004 and August 1^st^ 2006. Detailed methods are described elsewhere.[8]

### Data source

All 98 hospitals in the Netherlands with a maternity unit participated in this study. Monthly, local coordinators reported all cases using standardized web-based forms. Inclusion criteria were one or more of the following during pregnancy, delivery or puerperium: ICU/CCU admission; uterine rupture; eclampsia; major obstetric haemorrhage; other rare severe maternal morbidity to the opinion of the treating clinician. In the respective hospitals, cases were identified using maternity computer databases, labour ward diaries, staff reports, intensive care admission registers, blood transfusion registers, admission and discharge letters, letters concerning preconceptional advice and personal communication. Obtained data were made anonymous and consisted of a case record form with photocopies of relevant parts of the patient files such as those mentioned above.[8]

### Sample selection

From this cohort (n = 2552), the following women were selected from the database for the current study: 1) women admitted to intensive care unit (ICU) or coronary care unit (CCU) for cardiovascular disease during pregnancy, delivery and puerperium (limited to six weeks); 2) women on the ward with serious maternal morbidity from cardiovascular disease according to the treating physician, but not requiring ICU or CCU admission; 3) women who died before reaching ICU or CCU because of cardiovascular disease. Cases in which cardiovascular disorders were secondary to other complications (i.e. cardiac arrest after major pulmonary embolism) were excluded. All cases were discussed by the authors and placed in one of the categories when definitions were met. Women with pre-existing cardiovascular disease who had a normal, uneventful pregnancy, delivery and puerperium were not eligible for inclusion and remain beyond the scope of this study.

Cardiovascular disorders were categorized as: cardiomyopathy, valvular disease, ischaemic heart disease, arrhythmias and aortic dissection. Congenital heart disease was not a separate category due to small numbers and women were placed into the most appropriate category.

Peripartum cardiomyopathy (PPCM) was defined, according to Sliwa et al., as ‘an idiopathic cardiomyopathy presenting with heart failure secondary to left ventricular (LV) systolic dysfunction towards the end of pregnancy or in the months following delivery, where no other cause of heart failure is found. It is a diagnosis of exclusion. The LV may not be dilated but the ejection fraction (EF) is nearly always reduced below 45%’. [9] Hypertrophic cardiomyopathy was defined as echographically proven hypertrophied, non-dilated left ventricle in the absence of another systemic or cardiac disease that is capable of producing the same magnitude of wall thickening. Dilated cardiomyopathy was defined as an EF of <40% in the presence of increased left ventricular dimension, without other cause. Where possible, cases of arrhythmia were further specified as supraventricular tachycardia (SVT) or ventricular tachycardia (VT). SVT was defined as any tachycardia that was not ventricular in origin (ectopic atrial tachycardia, atrial fibrillation, atrial flutter and junctional tachycardia). VT was defined as tachycardia with ventricular origin, based on a 12-lead electrocardiography (ECG). Valvular disease was defined as cardiac valve stenosis or regurgitation when the diagnosis was made upon a heart murmur and symptoms in combination with echocardiographic abnormalities. Valvular disease was further specified into CHD, rheumatic disease or endocarditis based on the origin of valvular dysfunction. Ischaemic heart disease was defined as maternal complaints of chest pain accompanied by either rise of cardiac markers or ECG changes. Acute coronary syndrome was proven by raised cardiac markers (troponin >0.3 ug/l or CK-MB>170 U/l) in combination with complaints or ECG abnormalities. ST-depressions are known to occur rather frequently during caesarean section.[10] These mild ECG abnormalities in the absence of any abnormal clinical or laboratory findings were considered mild maternal morbidity and hence not included in the LEMMoN study. Sudden Arrhythmic (or Adult) Death Syndrome (SADS) is defined as sudden death in an adult for which no cause could be found.

Maternal deaths reported during the study period were cross-checked with the national Maternal Mortality Committee of the Dutch Society of Obstetrics and Gynaecology (NVOG). All maternal deaths due to cardiovascular disease were included. Results of post-portem investigations were collected, when available.

Maternal characteristics such as age, socio-economic status, body mass index (BMI), geographical ethnic origin and parity were collected, as well as all data on pregnancy and delivery and puerperium, including complications. Medical history was scrutinized for congenital heart disease, defined as structural or functional abnormality of the heart or intrathoracic great vessels that is present at birth and acquired heart disease, defined as a structural or functional abnormality of the heart that is acquired later in life. Cardiac risk factors such as smoking, hypertension, diabetes, hypercholesterolaemia (defined as total cholesterol ≥6.5 mmol/l) and a positive family history of cardiac disease under 60 years of age were identified.

### Outcome definitions

Main outcome measures were incidence of cardiovascular disorders and their respective CFRs.

Incidence was calculated using the total number of births in the Netherlands during the study period as denominator. Denominator data for the number of deliveries in the Netherlands were obtained from Statistics Netherlands (CBS) and based on birth registries after correction for stillbirths after 24 weeks of gestation and multiple pregnancies.[11] CFRs were calculated by dividing the number of deaths due to a specific condition by the total number of women with severe morbidity from that specific condition.

Possible risk factors for cardiac disorders in pregnancy were identified by calculating relative risks (RR) and 95% confidence intervals (CI) compared to the general pregnant Dutch population. National reference values, serving as the ‘comparison group’ were obtained from Statistics Netherlands and the Netherlands Perinatal Registry (LVR), a national registry containing outcome information of all pregnancies and deliveries from both midwives, obstetricians and general practitioners.[12]

### Statistical analyses

The data for the comparison group that was obtained from the LVR were used as reference values for calculating relative risk factors. Relative risks and confidence intervals were calculated in univariable analysis. Because the data only provided characteristics of all maternities together, we could not adjust relative risks for confounding variables, nor was a multivariable analysis possible. Differences between groups were identified using Chi square test, significance was defined as p<0.05. Statistical analyses were performed using Statistical Package for Social Sciences, version 17.0 (SPSS Inc, Chicago, IL, USA). The medical ethics committee of Leiden University Medical Center approved the LEMMoN study (P04-020; 8 March 2004).

## Results

All 98 maternity hospitals in the Netherlands reported a total of 2275 ‘hospital months’. Due to delayed participation of some hospitals, the LEMMoN study represented 97% of all deliveries in the study period (358,874 deliveries). Of the 2552 cases of severe maternal morbidity in the LEMMoN study, 84 (3.3%) involved cardiovascular disease. The incidence was 2.3 per 10,000 deliveries in the Netherlands.

There were 11 cases of maternal death due to cardiovascular disease, an incidence of 3.0 per 100,000 deliveries. CFR among women with severe morbidity from cardiovascular disorders of pregnancy was 13.1% (11/84), with nearly half of maternal deaths being caused by aortic dissection (Table 1). Of the 11 maternal deaths, one woman was known with congenital valvular disease, one with Marfan syndrome and one with hypertension. There were three maternal deaths among the 28 women with pre-existing acquired or congenital heart disease that experienced severe morbidity during pregnancy, delivery and puerperium (CFR 11%). Five women died during pregnancy, the other six postpartum.

**Table 1:** Types of cardiovascular disease and its mortality.

Type of cardiovascular disease	Morbidity n (% of total)	Mortality n (CFR)
**Cardiomyopathy**	**23 (27.4)**	**2 (9%)**
PPCM	17	1
hypertrophic CM	1	
dilated CM	2	
other, unspecified, pre-existing CM	3	1
**Valvular Disease** *	**19 (22.6)**	**0**
CHD	4	
rheumatic heart disease or fever	4	
endocarditis	1	
mechanical or biological valve	5	
other valvular disease	5	
**Ischaemic heart disease**	**17 (20.2)**	**1 (6%)**
General ischaemia	7	
ACS	10	1
- related to ritodrine (Pre-par®)	3	
- coronary artery dissection	1	
**Arrhythmias**	**16 (19.0)**	**1 (6%)**
SVT	11	
VT	5	1
**Aortic dissection**	**6 (7.1)**	**5 (83%)**
**Miscellaneous**	**3 (3.6)**	**2 (67%)**
pericarditis	1	
SADS	2	2
**Total**	**84 (100)**	**11 (13%)**

* more than one subcategory possible. PPCM: peripartum cardiomyopathy; CM: cardiomyopathy; CHD: congenital heart disease; ACS: acute coronary syndrome; SVT: supraventricular tachycardia; VT: ventricular tachycardia; SADS: Sudden Arrhythmic Death Syndrome.

Pre-existing acquired or congenital heart disease was identified in 34% of women. Most cardiovascular disease during pregnancy, delivery and puerperium thus occurred in women without pre-existing cardiac disease. Anticoagulants or medication for arrhythmias and/or hypertension before and/or during pregnancy was used by 20% of all women. Information concerning cardiac risk factors and previous (cardiac) history was available in 70% of women. Thirty-one percent of the women with cardiovascular disease were over the age of 35 yrs at the moment of inclusion and nearly 5% over the age of 40 yrs.

At least one cardiac risk factor of smoking, hypertension, overweight or obesity, diabetes, hypercholesterolaemia, or a family member with an acute coronary syndrome at a young age was present in 39% of the women. Of the women with a known BMI, 19% were overweight, 9% obese and 10% morbidly obese (Table 2).

**Table 2:** Characteristics of women with severe maternal morbidity caused by cardiovascular disease.

Characteristic	(PP)CM n = 23	VALVE n = 19	IHD n = 17	ARR n = 16	AD n = 6	MISC n = 3	TOTAL n = 84	%
**Age (mean 31.5)**								
<20 year	1	1	0	0	0	0	**2**	**2.4**
20–34 year	13	12	12	13	3	3	**56**	**67**
35–39 year	8	5	3	3	3	0	**22**	**26**
≥40 year	1	1	2	0	0	0	**4**	**4.8**
**Socio-economic status indicator**								
Low	5	11	5	3	1	0	**25**	**36**
Middle	12	4	5	7	3	3	**34**	**49**
High	3	2	2	3	1	0	**11**	**16**
Unknown	3	2	5	3	1	0	**14**	
**Smoking during pregnancy**								
Yes	0	0	2	1	0	2	**5**	**9.8**
No	12	10	12	10	2	0	**46**	**90**
Unknown	9	9	3	5	4	1	**31**	
**Body Mass Index (kg/m2)**								
<18.5	0	1	0	1	0	1	**3**	**5.1**
18.5–24.9	10	8	6	7	2	1	**34**	**58**
25–29.9 (overweight)	3	3	2	2	1	0	**11**	**19**
30.0–34.9 (obese)	2	0	1	2	0	0	**5**	**8.5**
≥35 (morbidly obese)	4	0	1	0	1	0	**6**	**10**
Unknown	4	7	7	4	2	1	**25**	
**Geographical ethnic origin**								
Western	18	9	14	10	6	3	**60**	**71**
Non-Western	5	10	3	6	0	0	**24**	**29**
**Prior Heart Disease** *								
Yes	5	14	2	5	2	0	**28**	**34**
Acquired	3	10	1	4	2	0	**20**	
Congenital	2	4	1	1	0	0	**8**	
No	19	5	15	10	3	3	**54**	**66**
Unknown	0	0	0	1	1	0	**2**	
**Prior Cardiac Medication** ∧								
none	19	11	15	13	6	3	**67**	**80**
diuretic	1	2	0	0	0	0	**3**	**3.6**
anti-arrhythmic or β-blocker	3	4	2	2	0	0	**11**	**13**
anti-coagulant	3	5	0	0	0	0	**8**	**9.5**
ACE inhibitor	1	0	0	1	0	0	**2**	**2.4**
**Comorbidity**								
*Diabetes*								
Type I	1	2	0	0	0	0	**3**	**3.6**
Type II	0	0	1	0	0	0	**1**	**1.2**
Gestational diabetes	1	0	0	0	0	0	**1**	**1.2**
No	21	17	16	16	6	3	**79**	**94**
*Hypertensive disease*								
(pre-)eclampsia	9	5	1	1	0	0	**16**	**19**
pregnancy induced	2	0	0	2	1	0	**5**	**6**
pre-existing	1	2	2	0	1	0	**6**	**7.1**
No	10	13	14	13	5	3	**58**	**69**
*Positive family history of heart disease*								
Yes	3	1	0	1	0	0	**5**	**10**
No	14	10	10	6	0	0	**40**	**83**
Unknown	6	8	7	9	6	0	**36**	

* not including pregnancy-induced hypertension or (pre) eclampsia,

∧ more than one option possible. (PP)CM: (peripartum) cardiomyopathy, VALVE: valvular disease, IHD: ischaemic heart disease, ARR: arrhythmias, AD: aortic dissection, MISC: miscellaneous.

In 8% of cases, the cardiovascular disorder occurred in early pregnancy, in 46% antepartum, in 10% during labour or caesarean and in 36% postpartum. Mode of delivery is shown in Table 3.

**Table 3:** Obstetric history and mode of delivery in women with severe maternal morbidity caused by cardiovascular disease.

Characteristic	(PP)CM n = 23	VALVE n = 19	IHD n = 17	ARR n = 16	AD n = 6	MISC n = 3	TOTAL n = 84	%
**Obstetric history**								
prior caesarean section	2	4	3	3	1	0	**13**	**15.5**
parity 0	16	10	9	8	2	2	**47**	**56.0**
parity 1–2	5	7	7	8	4	1	**32**	**38.1**
parity ≥3	2	2	1	0	0	0	**5**	**6.0**
**Current pregnancy**								
singleton pregnancy	20	17	16	16	4	3	**76**	**90.5**
multiple pregnancy	3	2	1	0	2	0	**8**	**9.5**
artificial reproduction techniques: IVF/ICSI	1	0	0	0	1	0	**2**	**2.4**
**Delivery**								
spontaneous delivery	4	5	5	8	0	0	**22**	**26.2**
ventouse/forceps delivery	2	2	2	1	0	0	**7**	**8.3**
induction of labour	6	6	2	2	0	0	**16**	**19.0**
pre-labour caesarean section	13	7	2	4	3	1	**30**	**35.7**
caesarean section overall	17	10	9	5	3	2	**46**	**54.8**
breech presentation	3	1	1	1	0	0	**6**	**7.1**
preterm birth (<37 w)	13	10	3	3	2	1	**32**	**38.1**
post term birth (≥42 w)	2	0	1	0	0	0	**3**	**3.6**
ICU or CCU admission	23	16	14	14	2	1	**70**	**83.3**

(PP)CM: (peripartum) cardiomyopathy, VALVE: valvular disease, IHD: ischaemic heart disease, ARR: arrhythmias, AD: aortic dissection, MISC: miscelaneous.

Comparing possible risk factors for developing cardiovascular disorders with reference to national data are shown in Table 4. [11]–[14] Associated factors in univariable analysis were caesarean section (RR 8.2, 95% CI 5.6–12.9), multiple pregnancy (RR 6.0, 95% CI 2.7–12.8), prior caesarean section (RR 2.9, 95% CI 1.5–5.4), non-Western ethnicity (RR 2.4, 95% CI 1.4–3.9) and obesity (RR 2.3, 95% CI 1.1–4.6).

**Table 4:** Comparison of possible risk factors for developing severe maternal morbidity and mortality caused by cardiovascular disease between cases and the general pregnant population.

	Cases n = 84	Netherlands, general pregnant population n = 358,874	
*Possible risk factor*	(%)	(%)	RR (95% CI)
**patient**			
age ≥35 years	31.0	24.7**	1.4 (0.8–2.2)
age ≥40 years	4.8	3.4**	1.4 (0.4–3.9)
BMI <18.5 kg/m^2^	5.1	3.1**	1.7 (0.4–5.5)
BMI ≥25 kg/m^2^ (overweight)	37.3	31.7**	1.3 (0.7–2.2)
BMI ≥30.0 kg/m^2^ (obese)	18.6	9.8**	**2.3 (1.1–4.6)**
BMI ≥35 kg/m^2^ (morbidly obese)	10.2	n/a	
low income	35.7	n/a	
non-Western immigrants	28.6	16.8	**2.4 (1.4–3.9)**
smoking during pregnancy	9.8	n/a	
single household	2.4	n/a	
chronic disease in history*	39.3	n/a	
**pregnancy**			
prior caesarean section	15.5	6.0^13^	**2.9 (1.5–5.4)**
multiple pregnancy	9.5	1.7**	**6.0 (2.7–12.8)**
parity 0	56.0	45.2**	**1.5 (1.0–2.4)**
parity ≥3	6.0	5.0**	1.2 (0.4–3.1)
artificial reproduction techniques: IVF/ICSI	2.4	1.9^14^	1.2 (0.2–5.1)
**delivery**			
ventouse/forceps delivery	8.3	8.6***	1.0 (0.4–2.2)
induction of labour	19.0	12.5***	**1.8 (1.0–3.1)**
pre-labour caesarean section	35.7	5.9***	**9.0 (5.6–14.4)**
caesarean section overall	54.8	13***	**8.2 (5.6–12.9)**
breech presentation	7.1	4.9***	1.5 (0.6–3.6)
preterm birth (<37 w)	38.1	5.8***	**10.3 (6.5–16.3)**
post term birth (≥42 w)	3.6	4.3***	0.8 (0.2–2.7)

n/a = data not available. RR = relative risk (95% confidence interval) **significant**.

* includes hypertension, diabetes, cardiac disease and coagulation disorders. National reference values from.

** [11] Statistics Netherlands (exact study period) and.

*** [12] The Netherlands Perinatal Registry (LVR-2, 2005).

Of the 84 women with cardiovascular disease, 70 (83%) were admitted to ICU or CCU. Five women were being resuscitated or already dead upon arrival in hospital.

Preconceptional advice was given in 21% (6/28) of women with pre-existing cardiac disease. It remained unclear whether or not preconceptional advice was given for 9 women. There was a high index of suspicion of cardiac disease in 14% (12/84) of women. In these cases, either patient (n = 3) or doctors delay (n = 9) may have played a role in the progression of adverse outcomes.

Multidisciplinary consultation between obstetricians and cardiologists or anaesthesiologists took place in 86% (32/37) of women with antepartum cardiac disease. Most important topics were expected tolerance of cardiac strain during labour and endocarditis prophylaxis. Measures agreed upon included endocarditis prophylaxis (n = 9), primary epidural analgesia (n = 5), telemetry during delivery (n = 4), termination of pregnancy (n = 2) and primary instrumental delivery (n = 2).

During the study period, 19 women experienced cardiac arrest with a CFR of 63%. Nine of them were included in the current study group with primary cardiovascular disease. These had cardiac arrest preceded by aortic dissection (n = 4), arrhythmia (n = 2), SADS (n = 2) and spinal anaesthesia in combination with pre-existing cardiomyopathy (n = 1). Excluded were those that had cardiac arrest preceded by non-primary cardiovascular disease.

### Cardiomyopathy

Twenty-three women experienced cardiomyopathy, including 17 women with PPCM. Among the 12 antepartum cases, mean onset of disease was at 34 weeks gestation. Ten of the 17 women with PPCM either had pregnancy induced hypertension (PIH), pre-eclampsia (PE) or HELLP syndrome. Six women admitted to the ICU or CCU were intubated for mechanical ventilation followed by emergency caesarean section. Two women developed terminal heart failure requiring bridge-to-transplant therapy (heartmate, left ventricular assist device) followed by an Implantable Cardioverter Defibrillator (ICD) in one woman and heart transplant in the other. There were two maternal deaths (CFR 8.7%): both due to postanoxic encephalopathy, the first after cardiogenic shock despite introduction of an intra-aortic balloon pump and the other after ventricular fibrillation and cardiopulmonary resuscitation (CPR). Complications occurred after major obstetric haemorrhage in two women.

### Valvular disease

Nineteen women developed symptomatic valvular disease with two-thirds (13/19) presenting antepartum with median onset at 30 weeks gestation. The four women with valvular disease based on previous acute rheumatic fever were all from Mediterranean descent. Three had mitral valve stenosis and one suffered from aortic stenosis. Four other women had aortic stenosis or aortic-, mitral- or tricuspid regurgitation based on CHD. There were five women with cardiac valve prostheses including two biological and three mechanical prostheses. All three women with mechanical prostheses developed thromboembolic complications, two after anticoagulant therapy switch and one without therapy switch. Two women with a biological prosthetic heart valve developed arrhythmia after mild postpartum haemorrhage. Two of the five women who were admitted antepartum subsequently delivered at the ICU or CCU. Although there was no maternal death in this category, there was significant long-term morbidity. Three women developed heart failure, three arrhythmias and one woman with a mechanical valve developed both. Two women with arrhythmia received direct current cardioversion several weeks postpartum. One woman with secondary heart failure required mitral and aortic valve surgery six weeks postpartum.

### Ischaemic heart disease

Seventeen women developed ischaemic heart disease (IHD), including ten women with acute coronary syndrome (ACS). Mean onset of disease was almost exclusively intrapartum or directly postpartum at a median gestation of 40 weeks. IHD developed after the use of ritodrine (Pre-Par®) in three women. Complications occurred after major obstetric haemorrhage in five women, four of whom had been given sulproston (Nalador®). Treatment or diagnostic modalities included percutaneous transluminal coronary angioplasty (PTCA) (n = 3), coronary angiography (n = 2) and drug administration (n = 3). There was one maternal death (CFR 5.9%). She was admitted to the CCU six weeks postpartum in cardiogenic shock. Despite CPR and PTCA, death occurred due to dissection of the left anterior descending coronary artery as confirmed at postmortem.

### Arrhythmias

Sixteen women experienced cardiac arrhythmias, including 11 SVTs and 5 VTs. SVTs included Wolff-Parkinson-White syndrome (n = 2), atrial fibrillation (n = 2), after major obstetric haemorrhage (n = 3) or were not further specified in the other four women. Onset of disease was mostly antepartum (n = 9) with a median onset at 33 weeks gestation. There was one maternal death (CFR 6.3%) eight days postpartum after cardiac arrest based on ventricular fibrillation for which cardiac catheterization and insertion of an intra-aortic balloon pump was performed. Two women with ventricular tachycardia required ICD implantation, which was placed postpartum. Pharmacological or electrical cardioversion was performed in seven and five women, respectively.

### Aortic dissection

Six women had aortic dissection, five of whom died (CFR = 83%). Three women died before delivery and two shortly after emergency caesarean section. Presentation was at a median gestation of 34 weeks. At 24 weeks gestation, ascending aorta dilation measured 57 mm in a woman with Marfan syndrome. She died five days postpartum during emergency surgery for aortic dissection due to pulmonary artery rupture.

### Miscellaneous

A primiparous woman was admitted at 33 weeks gestation with hemolysis, elevated liver enzymes, low platelets (HELLP) syndrome, progressive acute respiratory distress syndrome and pneumonia. After emergency caesarean section, her ECG was indicative of pericarditis secondary to pneumonia.

SADS occurred in two women. One perimortem caesarean section was performed in the delivery room in a term multiparous woman after acute collapse. Postmortem investigation did not reveal exact cause of death but did show goiter (91 grams) and congested hepatomegaly (2320 grams). There were no signs of acute coronary syndrome or lung or amniotic fluid embolus. Based on the clinically seen acute cyanosis and congested liver, the cause of death was considered of cardiac origin. Another primiparous woman was found at 23 weeks gestation and died after resuscitation. Postmortem investigation showed fibrosis of the interstitial wall of the left ventricle and all organs showed extreme hyperaemia, indicating possible terminal heart failure. Traces of cannabis were also found, although this was of unsubstantial amount to explain this death. Based on these findings, cardiac origin as a cause of death was most likely.

## Discussion

In this study we report all severe cardiac disorders of pregnancy and childbirth in a two-year nationwide cohort. Most severe maternal morbidity from cardiac disorders occurs in women without pre-existing cardiac disease. Our incidence of severe maternal morbidity from cardiovascular disease of 2.3 per 10,000 deliveries is much lower than the ratios of 0.2–4% presented in the ESC Guidelines on the management of cardiovascular disease during pregnancy by the Task Force of the European Society of Cardiology (ESC).[15] The Netherlands, considered a developed country, may have possibly better outcomes compared to developing countries.[16] However, the ESC percentages concern pregnancies complicated by cardiovascular disease, not *severe* maternal morbidity as was the aim of our study. For example, women with pre-existing disease who did not experience severe maternal morbidity were not captured in this study. Women with pre-existing congenital or acquired cardiovascular disease that developed severe maternal morbidity represented 34% of this cohort, half of them concerning women with arrhythmias.

Other studies have found that maternal mortality due to cardiovascular disease has increased over the last decade. It can be assumed that severe morbidity due to cardiovascular disease is also increasing. This coincides with cardiac risk factors such as increasing maternal age and obesity incidence in the general Dutch population. Another determinant may be the fact that more women with CHD reach childbearing age due to advances in treatment. Cardiovascular complications are reported in 11% of pregnancies in women with different types of CHD.[17] A prospective follow-up study of all women with CHD is currently being performed in the Netherlands under the name of ‘ZAHARA-II’.[18]

The increase in maternal deaths from cardiovascular disease in the Netherlands is similar to findings in the UK.[7] In the Netherlands, the mean age at first pregnancy in 2006 was 29.4 years of age, a five-year increase compared to 1970. In 2006, 7.0% of women had their first pregnancy after their thirty-sixth birthday, compared to only 2.3% in 1970.[19] Increasing maternal age and in addition an unhealthy cardiovascular lifestyle have brought along an increased prevalence of chronic medical conditions such as obesity, hypertension and diabetes and concomitantly more cardiovascular complications such as acute coronary syndrome and (pre)eclampsia during pregnancy.[20]

The strength of this study is that cardiovascular disorders were extracted from the first nationwide study of severe maternal morbidity in the Netherlands and to date, the largest prospective study in literature. This prospective design makes this study unique but comparison with other studies unsound. Before relating our results to those of other countries, it should be noted that these results might not be directly comparable to the Dutch situation because of differences in baseline demographics, health and obstetric practices in our country. Especially the use of caesarean section is relatively low in our country and delivery at home is still common.

In the United States, a large National Hospital Discharge Survey identified severe maternal morbidity because of cardiac disease during delivery hospitalisations between 1991 and 2003 in 0.41 per 1000 deliveries and 6,7% of all SAMM as compared to 0.23 per 1000 deliveries and 3.3% of all SAMM in our study.[21]

Two other large European studies addressing overall incidence of severe maternal morbidity in Western countries did not separately specify cardiovascular disease.[22], [23] A large retrospective cohort in Canada, showed a significantly increased myocardial infarction rate from 0.00 to 0.02 per 1000 deliveries between the triennium ending in 1993 and in 2000 giving a RR of 3.70 (95% CI 1.21–11.35).[24]

In 11 of 84 cases (13%), severe adverse effects of drugs have likely played a role in our study. IHD and pulmonary oedema after the use of ritodrine (Pre-Par) has been described in several case reports.[25]–[28] In our study, three women experienced IHD and one experienced PPCM after the use of ritodrine. As oxygen consumption increases during pregnancy, addition of β-receptor stimulation, further increasing oxygen consumption, may induce IHD. IHD, coronary spasms and pulmonary oedema after the use of sulproston (Nalador) has also been described in several case reports.[29]–[31] In our study, i.v. administration of sulproston was associated with cardiac problems in 7 women: IHD (n = 4), PPCM (n = 1), SVT (n = 1), and mitral valve regurgitation (n = 1). However, the drug was administered after major obstetric haemorrhage in all seven cases, with a median blood loss of 2,5 litres (range 1,5 to 5 litres) and a median transfusion of five red blood cell units (range 4 to 10 units). It is uncertain if IHD was caused by the haemorrhage leading to hypovolemia with subsequent ischaemia or solely due to the administration of sulproston. In cases with relatively little blood loss, the complication is more likely caused by sulproston. Pre-existing cardiac disease is a strong contra-indication for administering these drugs, and awareness of cardiac side effects is necessary when administering these drugs even in the absence of cardiac disease. Even though the Dutch authority for drug administration contraindicates sulproston administration to women with cardiovascular disease, the drug was administered to one woman with known mitral valve insufficiency after major obstetric haemorrhage, albeit without subsequent complications.[32]

Peripartum cardiomyopathy was preceded by hypertensive disorders or pre-eclampsia in 10 of the 17 women giving an identifiable cause for heart failure. It seems important for the prognosis of future pregnancies to distinguish whether or not heart failure was preceded by pregnancy induced hypertensive disorders as a cause for PPCM.[33]

Overall, cardiovascular complications occurred after major obstetric haemorrhage in 13 women. Within the LEMMoN-study 13 of the 1590 women (0.8%) with major obstetric haemorrhage developed cardiac complications. Although not primarily a cardiac complication, hemodynamic changes of pregnancy may cause inadequate hemodynamic compensation for major haemorrhage leading to severe cardiac complications.

The diagnosis of cardiomyopathy is more difficult in pregnancy because of a more extensive differential diagnosis, including normal left ventricular dilatation during pregnancy, amniotic fluid embolism, pulmonary embolism, pre-eclampsia and placental abruption. Amniotic fluid embolism or pulmonary embolism was considered in three and seven women with acute dyspnoea, respectively.

Vascular dissections including aortic and coronary dissections were the main contributor to maternal mortality by cardiovascular disease in our study and are further described by la Chapelle et al. over a period of 15 years in the Netherlands.[34]

Measures such as primary epidural anaesthesia with or without primary instrumental delivery, restriction of the duration of the second stage of labour and elective caesarean section were agreed upon by obstetricians and cardiologists or anaesthesiologists in order to reduce variations in blood pressure and thus cardiac strain. Cardiac monitoring during delivery was also warranted in specific cases.

Caesarean section as mode of delivery during this study was found to be a possible risk factor for cardiovascular disease. Of the 46 women who delivered by caesarean, 19 women developed cardiovascular disease during or after caesarean section. It is possible that caesarean sections, associated with hemodynamic fluctuations associated with intubation, analgesia and choice of anaesthesia and haemorrhage, aggravated maternal condition leading to cardiovascular disease. For example, epidural anaesthesia gives significant peripheral vasodilation with subsequent hypotension, which may trigger ischaemic heart disease. However, we can not exclude the possibility that more symptomatic patients have received caesarean sections because of their condition.

Non-Western immigrants comprise nearly a third (29%) of our cohort, compared with 17% percent of the general population. Non-Western immigrants thereby contribute substantially to the incidence of severe morbidity from cardiovascular disease, especially diseases preceded by rheumatic fever (n = 4). A general medical check up in recently immigrated women may signal risk factors or disease and improve maternal health through adequate, early, education.

Other high-risk groups are obese women, women with a prior caesarean delivery, women with multiple pregnancies and nulliparous women. Obesity is not only a cardiac, but also an obstetric problem and should be addressed by both national campaigns to improve general health as well as by counselling in early, or preferably before, pregnancy. Preventing a first caesarean delivery leads to less morbidity in subsequent pregnancies and opting for caesarean section should therefore be weighed carefully when the indication is not strict. In case of artificial reproduction, preventing multiple gestations is extremely important, leading to less maternal and neonatal morbidity and mortality. Physicians should have increased awareness for development of cardiovascular disease in these high risk groups and should hold a low threshold for adequate early pregnancy screening and referral to a cardiologist and/or anaesthesiologist early in pregnancy and at least prior to delivery.

Women with pre-existing cardiac disease should be referred for preconceptional advice, optimising preconceptional health and staying within scope throughout pregnancy in a specialised centre. As conditions may worsen, preconceptional advice is also indicated in women with pre-existing cardiac disease who have already had uneventful pregnancies and deliveries in the past. In our cohort, based on the available information from photocopied files, only a low percentage of women with pre-existing cardiac disease actually did have preconceptional advice. In our opinion, women with a cardiac family history and women who have had chemotherapy in childhood should also be eligible for preconceptional screening with echocardiography and advice afterwards.

Delay in treatment may be the result of numerous factors. First of all, the family history is often only partially known or told by the patient or not asked for in detail by the physician leading to inadequate information for risk assessment. Secondly, pregnant women, especially nulliparous, may attribute symptoms to pregnancy until they have aggravated so severely that adequate treatment is out of reach. In addition, symptoms may not always be taken seriously by health care providers, adding to a delay in diagnosis and treatment. For example, symptoms of shortness of breath and increasing fatigue may accompany uncomplicated pregnancy but are also symptoms of coronary heart disease. Sometimes, no clarifications for existing symptoms may be found despite adequate investigations, giving false reassurance. When there is a high index of suspicion, it is imperative that symptoms be taken seriously and pathology is excluded, and that women are told to contact their physician in case of recurrent or persisting symptoms. A delay in diagnosis or treatment may also have occurred due to communication difficulties among the Non-Western women concerning access to care.

The results of this study should increase awareness of the occurrence of severe maternal morbidity from cardiovascular disease and will hopefully lead to adequate risk assessment and timely referral of women at risk. The ZAHARA-II study group and the European Society of Cardiology are currently performing more research on pregnancy and acquired and congenital cardiovascular disease.

### Study limitations

The main limitation of this study is that we were dependent upon the selection of women and the information that was submitted by the local coordinating obstetrician. While these were all very dedicated abstractors, objective inclusion criteria were lacking therefore leaving inclusions of rare conditions of severe maternal morbidity as well as severe manifestations of generally less severe conditions up to the opinion of the treating obstetrician. These factors may contribute to bias and underestimation. Potentially missing cases most likely concern women with *less severe* morbidity, as opposed to those *with* severe morbidity. Morbidity is difficult to classify, with a wide range of severity all considered ‘morbidity’.

Although extensive general and obstetric information was usually present in every case, specific detailed cardiologist reports, ICU records or follow-up letters concerning cardiac risk factors were not complete in all cases. This sometimes complicated the process of classifying cases based on the correct diagnosis and could possibly skew the distribution of causes of cardiovascular disorders as seen in Table 1. Information on certain cardiac risk factors was missing such as smoking (31%) and BMI (25%).

Another limitation is that individual characteristics of all maternities without severe maternal morbidity during the study period were not available. Therefore, we could not adjust relative risks for confounding variables, nor was a multivariable analysis possible. Case ascertainment cannot be guaranteed but is considered accurate. Especially the most severe cases of cardiac complications are unlikely to be missed. Postpartum complications that occurred more remote from delivery, however, could have been missed as they might stay out of the scope of the obstetrician.

An important limitation is that women with pre-existing acquired or congenital heart disease who did not experience severe maternal morbidity were not captured in this study. This makes it impossible to calculate the chance of severe morbidity for all women with pre-existing acquired or congenital heart disease in general. It should therefore be noted that the mentioned CFRs is of limited use, as it is based on women with severe maternal morbidity from cardiovascular disease only.

### Conclusion

Cardiovascular disease is a rare cause of severe maternal morbidity with a high case fatality rate, especially in the case of aortic dissection. Most cardiac disorders in pregnancy, delivery or puerperium develop in women without pre-existing cardiac disease. In 13% of cases a serious adverse event of drugs might have played a crucial role in the development of the complication.
